# Discernment of transformer oil stray gassing anomalies using machine learning classification techniques

**DOI:** 10.1038/s41598-023-50833-7

**Published:** 2024-01-03

**Authors:** M. K. Ngwenyama, M. N. Gitau

**Affiliations:** Department of Electrical, Electronic and Computer Engineering, University of Pretoria, Pretoria, 0002 South Africa

**Keywords:** Electrical and electronic engineering, Power distribution, Power stations

## Abstract

This work examines the application of machine learning (ML) algorithms to evaluate dissolved gas analysis (DGA) data to quickly identify incipient faults in oil-immersed transformers (OITs). Transformers are pivotal equipment in the transmission and distribution of electrical power. The failure of a particular unit during service may interrupt a massive number of consumers and disrupt commercial activities in that area. Therefore, several monitoring techniques are proposed to ensure that the unit maintains an adequate level of functionality in addition to an extended useful lifespan. DGA is a technique commonly employed for monitoring the state of OITs. The understanding of DGA samples is conversely unsatisfactory from the perspective of evaluating incipient faults and relies mainly on the proficiency of test engineers. In the current work, a multi-classification model that is centered on ML algorithms is demonstrated to have a logical, precise, and perfect understanding of DGA. The proposed model is used to analyze 138 transformer oil (TO) samples that exhibited different stray gassing characteristics in various South African substations. The proposed model combines the design of four ML classifiers and enhances diagnosis accuracy and trust between the transformer manufacturer and power utility. Furthermore, case reports on transformer failure analysis using the proposed model, IEC 60599:2022, and Eskom (Specification—Ref: 240-75661431) standards are presented. In addition, a comparison analysis is conducted in this work against the conventional DGA approaches to validate the proposed model. The proposed model demonstrates the highest degree of accuracy of 87.7%, which was produced by Bagged Trees, followed by Fine KNN with 86.2%, and the third in rank is Quadratic SVM with 84.1%.

## Introduction

With the radical growth in the power system capacity, the demands for power generation, transmission, and distribution, have become greater^1^. As a significant piece of equipment for power distribution in power systems, the power transformer (PT) is critical for the secure operation of the complete power system. The occurrence of a fault in a PT will result in damage to the unit. The most severe faults might even cause the failure of the entire power system, adversely affecting the functioning of the total national economy. Thus, it is beneficial to examine fault diagnosis technology relating to PTs^2^. PT faults usually emerge from electrical and thermal stresses, such faults vary merely in their energy, site, and time of occurrence. The oil temperature increases and several gases will be generated when the fault occurs. Generally, the combustible gasses found in the TO in service are hydrogen $\left({\text{H}}_{2}\right)$, methane $\left({\text{CH}}_{4}\right)$, ethane $\left({\text{C}}_{2}{{\text{H}}}_{2}\right)$, ethylene $\left({\text{C}}_{2}{{\text{H}}}_{4}\right)$, and acetylene $\left({\text{C}}_{2}{{\text{H}}}_{6}\right)$^3,4^. The pollutants in oil are mostly the consequence of the degradation of insulating elements (oil or sheet) because of faults or chemical responses in the apparatus in question.

The quality and quantity of disintegrated gases have a prominent function in assessing the fault type in PTs^5,6^. Many conventional techniques have been developed to analyze transformer faults with gas chromatography; a procedure where a chemical combination transported through a gas or liquid is broken down into its constituent parts as a result of the substances flowing differently along or above a static solution. Such schemes for fault analysis are usually categorized into three types, specifically, the distinctive gas scheme^7–10^, the gas production rate scheme^10^, and the three-ratio scheme^11–13^. In China, over 50% of the PT faults in the energy system were evaluated by employing DGA-based analysis schemes which analyze transformer fault types and their severity following the content, proportion to one another, and the gas production rate of the DGs in the TO^13^. Adding to the above three key conventional techniques, some enhanced schemes have emerged, like the Doernenburg scheme, the Rogers ratio scheme, the Duval triangle scheme, the International Electrotechnical Commission (IEC) ratio scheme, and the Key Gas (KG) scheme^14–18^. Such schemes usually employ numerous gas ratios or compare gas levels with the appointed criteria to analyze the state of a PT. However, most of these conventional analysis techniques provide a restricted impact to a transformer’s fault analysis, which is unable to precisely identify its correct fault type. Particularly, it is extremely complex to precisely determine the fault state with several DGs, a great probability of misdiagnosis will occur when the calculated and analyzed gas ratio is near the critical value^19^. Furthermore, the more comprehensive the classifications of fault types are, the lesser the precision rate of fault analysis is, and vice versa. Moreover, rough classifications are not conducive to the fault analysis of a PT, and it is challenging to meet the demands of applications.

**Figure 1:**
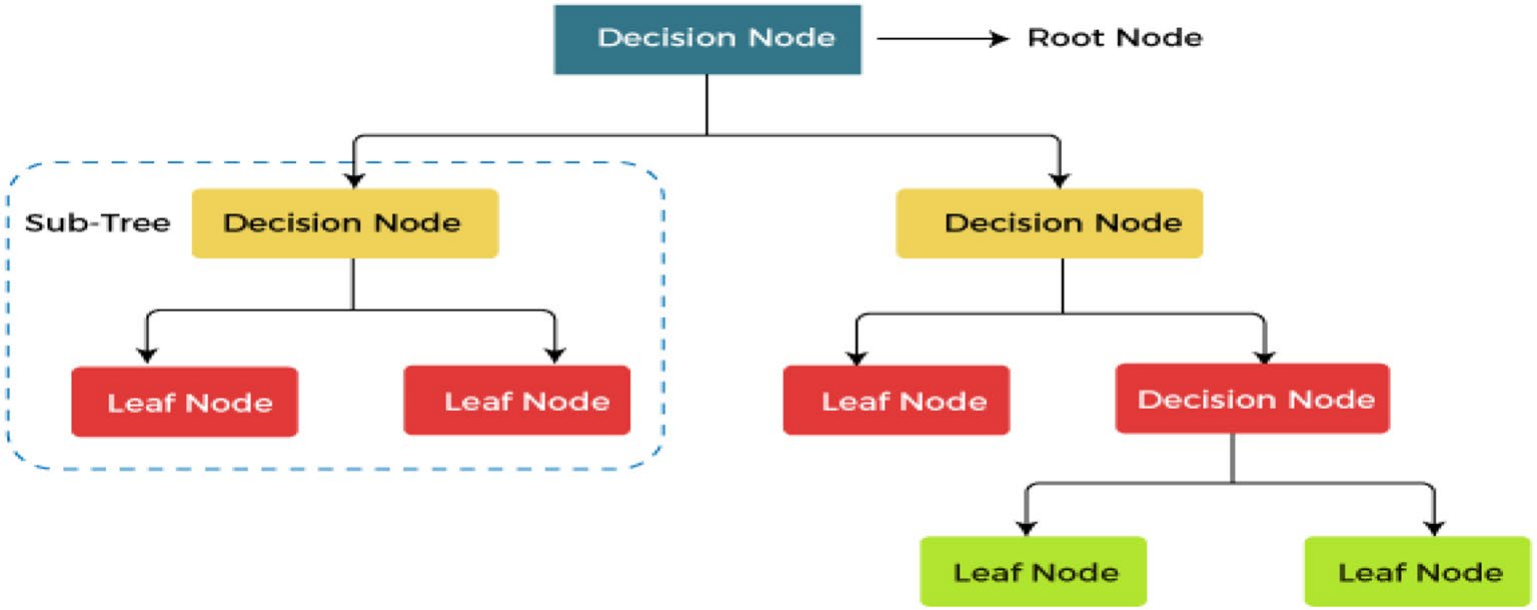
Example of DT.

**Figure 2:**
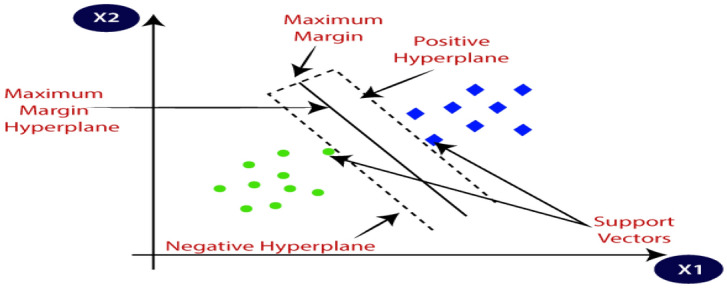
Example of SVM.

**Figure 3:**
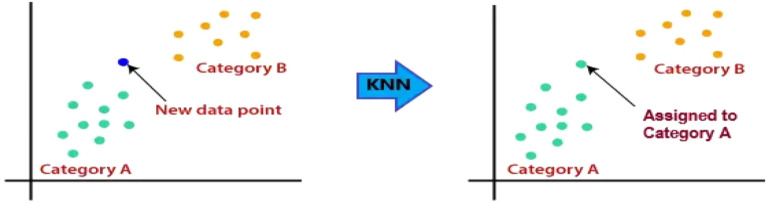
Example of KNN.

**Figure 4:**
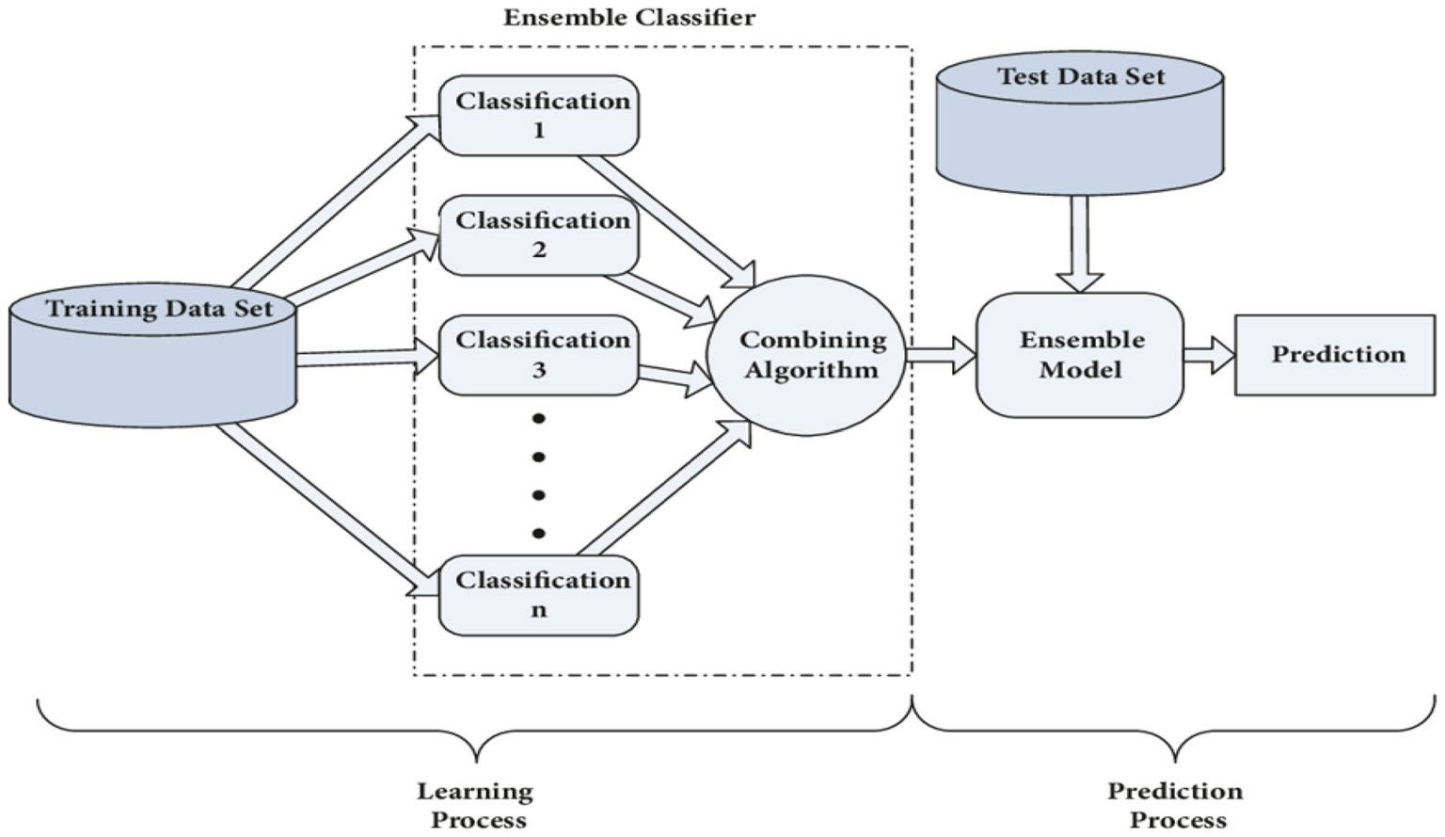
Example of EC.

DGA is a technique for detecting and forecasting problems in OITs by (i) determining the levels of various gases contained in the insulation oil, as well as respective gas rates and gas proportions, (ii) fault detection utilizing diagnosis instruments such as KG^20,21^, IEC ratios^22^, Rogers ratios^23^, Doernenburg ratios^24^ and Duval triangle^23^. Nevertheless, these instruments have certain flaws. In certain situations, the computed gas ratios deviate from the instruments’ specified ratio codes. Faults that develop within the transformer might be undetectable^25^. Additionally, these instruments can produce various analytical outcomes for the equivalent dissolved gas (DG) file, making it challenging for experts to reach a definitive conclusion when confronted with such a wide range of data^26^. Due to these constraints, several scientists have developed systems that are integrated with ML approaches that use historical DGA information to forecast imminent or undiscovered faults for diagnosing faults. The complexity of identifying the appropriate fault situation and the analytic precisions for units under fault categories are defined by these aspects^27,28^. The KG ratios, as well as graphic depiction schemes, are all DGA schemes that are utilized as data inputs to ML classifiers for fault classification. In the current study, a multi-classification model that is centered on ML algorithms is shown to have an intelligible, precise, and clear understanding of DGA. This enthusiasm is supported by (i) efficient adaptation to fresh data in ML; (ii) for structural layout, ML needs minimal exertion (i.e. several control settings are involved.); and (iii) the capability of ML to categorize unpredictable issues^29^. Capitalizing on these benefits, the proposed model is used to analyze and evaluate the state and suitable gas name subscription of 138 TO samples that exhibited different stray gassing characteristics in various South African substations. The model uses four ML classifiers, namely: (i) Decision Tree (DT)^30^; (ii) Support Vector Machine (SVM)^31^; (iii) K-Nearest Neighbour (KNN)^32^; and (iv) Ensemble Classifier (EC)^33^. These classifiers are applied for oil sample classification and are selected based on their capacity to compare new data inputs to existing data to identify the class that closely resembles existing classes to place new data within. In MATLAB/Simulink, the proposed model serves as the framework underlying the various classifiers and is designed to aggregate ML algorithms for information-gathering activities. A detailed summary of the various ML classifiers utilized in this work is provided in the section that follows:

- DT: As shown in Fig. 1, the DT classifier^34^ is an ML technique that makes predictions using a tree structure. It builds a flowchart-like tree structure where each internal node represents a feature test, each branch represents a test outcome, and each leaf node stores a class label. It is constructed by constantly splitting the training data into subsets depending on feature values until a stopping requirement is met, such as the maximum depth of the tree or the minimum number of samples needed to divide a node. The method replicates the operation for every split subgroup that is the offspring of a given node. Lastly, the tree is trimmed by deleting limbs that are not useful for classification.
- SVM: The working of the SVM classifier^35^ can be understood by using Fig. 2. SVMs fall within the broad group of kernel schemes^36^ that rely solely on data using mark pairings. To guarantee that the hyperplane is as broad as feasible across categories, the kernel function determines an estimation product for certain potentially large-scale feature regions. SVMs possess the benefits of becoming less mathematically intensive compared to different methods of classification, performing well in large-scale areas, as well as managing unpredictable classification effectively by utilizing the kernel trick, which subsequently converts the data area into a different large-scale feature area.
- KNN: The KNN classifier^37^ is a monitored learning approach utilized for numerous machine learning scenarios. It arranges elements using the nearest trained samples in the characteristic domain. The goal underlying KNN is to locate a well-known amount of training data that is nearest in proximity to a particular querying case and estimate the querying case's category based on them. Regarding categorization, KNN is comparable to a DT method, except that rather than developing a tree, instead, it creates a route through the graph. KNNs are also quicker compared to DTs. The working of the KNN is shown in Fig. 3.
- EC: The ensemble classifier^38^ produces classification forecasts using a set of classifiers, which achieves more accurate specialization than one classifier and results in an improved measurement grade. A dataset is used to train a list of classifiers, and the separate predictions made by each of the classifiers applied to the dataset form the basis of EC. The ensemble model then combines the outcomes of each classifier prediction to get the final result. This sort of classifier remains simple to simulate but is often appropriate for large samples. The working of the EC is shown in Fig. 4.

**Figure 5:**
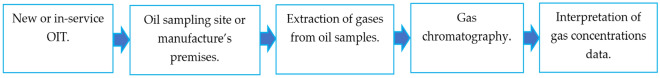
Extraction of transformer oil for DGA.

In monitoring the insulation status in OITs, several chemical and electrical processes are employed, such as DGA and Furan Analysis (FA), which indicate the Degree of Polymerization (DP) of the cellulose paper^6,39^. DGA is one of the most common methods for detecting an incipient fault in PTs. DGA can be used to assess present-day transformer status, predict future failures, and identify inconvenient transformer operations to provide appropriate maintenance planning. Figure 5 illustrates the standard technique employed by the transformer manufacturing sector to collect transformer oil on-site for DGA at the testing facility.

The presented DGA approaches do not contain any mathematical development, and the assessment depends on an experiential method that can vary depending on the expertise of the laboratory analyst, which results in unpredictable assessment^40^. To overcome this limitation, several computational models based on ML have been used in assessing incipient faults in PTs. In the proposed research work, recent related studies and their contributions to transformer fault diagnosis have been highlighted and a multi-classification model for transformer fault diagnosis is proposed. Table 1 presents a comparative study of the existing recent survey and the proposed model for transformer fault analysis.

**Table 1:** Summary of recent related studies.

Ref	Year	Proposed technique	Contribution
^41^	2018	Doernenburg ratio approach, Roger’s ratio approach, multi-layer ANN perceptron	A fault diagnostic analysis was performed by developing a hybrid Doernenburg and Rogers ratio technique to determine a gas ratio suitable to train a multi-layer ANN perceptron
^42^	2019	Mean Shift algorithm (MSA), ANN	An MSA-based ANN is proposed. The IEC 60,599:2007 standard consists of gases that are used to create parameters that will be trained using the proposed method. The MSA was used to effectively avoid the limitation of the number of training patterns (data size). The training and validation techniques both produced acceptable outcomes
^43^	2020	Fuzzy Logic, IEC ratio approach	A fuzzy logic-IEC ratio approach was proposed for transformer fault diagnosis. The outcomes demonstrate an improvement over the conventional IEC ratio technique
^44^	2022	ANFIS, Roger’s ratio approach	A hybrid Rogers ratio technique-based ANFIS was proposed to detect transformer faults. The training was carried out by employing the gas ratios presented by the IEEE C57-104 and IEC 60,599 standards
Current study	2023	Multi-classification model	A multi-classification model for fault diagnosis is proposed, that enhances diagnostic accuracy and optimism between transformer manufacturer and power utility Case reports on transformer fault analysis utilizing the proposed multi-classification model, IEC 60599:2022, and Eskom (Specification—Ref: 240-75661431) standards for fault analysis have been presented

### Contribution and novelty

#### Research contribution

This research work provided a summary of recent transformer fault analysis. Several ML-based techniques based on conventional DGA approaches have been discussed. The following are the contributions of the proposed research analysis:

- A multi-classification model for fault diagnosis is proposed, that enhances diagnostic accuracy and optimism between transformer manufacturer and power utility.
- Case reports on transformer fault analysis utilizing the proposed multi-classification model, IEC 60599:2022 and Eskom (Specification—Ref: 240-75661431) standards for fault analysis have been presented.

#### Research novelty

The main objective of the current research is to contribute to the practice of TO analysis. Although various current research studies have concentrated on TO analysis, minimal and occasional research has been published on the adoption of a multi-classification model, IEC 60599:2022, and Eskom (Specification—Ref: 240-75661431) standards for OIT analysis. The proposed model is a significant technique for overcoming the inadequacies of the IEC gas ratio technique to create an effective oil analysis tool. The seven fault categories utilized in the IEC 60599:2022 standard were considered and concluded that the degree of accuracy for fault detection is not ideal as a consequence of the limits defined by the gas ratio codes, and leads to "not detectable" in certain cases scenarios. However, after introducing the proposed model, the analysis is on an equal footing with the actual fault analysis. Furthermore, this research work addressed the optimal ratios of fault analysis. It is crucial to train the proposed model. Consequently, the DGA data utilized to train the proposed model is made up of samples that cover all known types of faults as defined by the IEC 60599:2022 standard. According to the findings of this research, the forecasting of transformer faults employing the proposed model as well as the IEC 60599:2022 gas ratio technique is comparable to actual fault analysis and offers an improvement over the IEC 60599:2022 gas ratio technique.

### Paper organization

The rest of the work is structured as follows: Section “Review of existing DGA approaches” provides an overview of current DGA approaches. Section “Applicable works” provides an overview of techniques employed by researchers to explore DGA. Section “Proposed approach” discusses the research approach and model. Section “Materials and protocols” presents the materials and protocols of the study. Section “Results” presents the results and discussions that validate the proposed model. and finally, Section “Conclusions” presents the conclusions of this work.

## Review of existing DGA approaches

**Figure 6:**
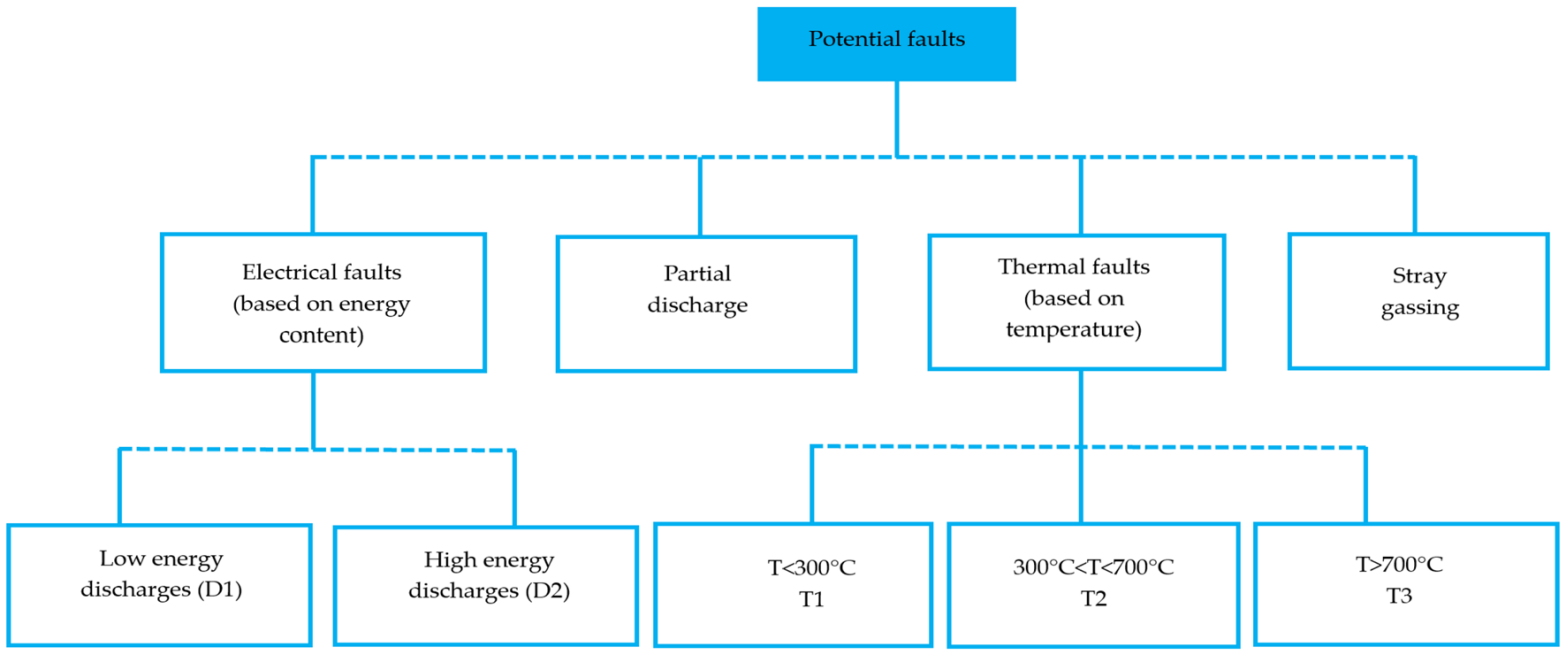
Dataset of potential faults^53^.

There are several procedures for diagnosing deformities in transformer insulation. DGA analysis strategies are dependent on scientific hypotheses and practical knowledge gained by specialists across the world^45,46^. However, if these analysis strategies are not implemented with caution, they might detect abnormalities erroneously since they simply signal potential faults^47^. DGA strategies can vary regarding diagnosed faults in several instances, which is undesirable for an accurate fault analytic technology^48^. Flammable and non-flammable gases can be discovered within the gases contained in the oil, as shown in Table 2. These gases can also be categorized based on the type of fault that induced them, as shown in Table 3. Gas levels, KGs, KG ratios, and graphic interpretations are mutual ideologies adopted in analysis strategies^49^. The DGA can recognize different faults such as partial discharge, excessive heat, as well as arcing in a wide range of PTs. A single dataset is required for the efficiency analysis and analytical comparison of conventional DGA approaches^50^. Figure 6 illustrates a dataset of potential transformer faults. Furthermore, DGA can give the timely detection required to maximize the probability of establishing a suitable remedy^51,52^. Numerous informative techniques based on DGA to identify the emerging fault type have been stated. In this work, seven of the DGA approaches were explored: (i) the CIGRE approach, (ii) the Doernenburg ratio approach, (iii) the KG approach, (iv) the Nomograph approach, (v) the IEC ratio approach, (vii) Duval triangle approach, and (viii) Rogers ratio approach.

**Table 2:** DGs in TO.

Gases	
Flammable	Non-flammable
Carbon monoxide (${\text{CO}}$)	Oxygen (${\text{O}}_{2}$)
Hydrogen (${\text{H}}_{2}$)	–
Methane (${\text{CH}}_{4}$)	Nitrogen (${\text{N}}_{2}$)
Ethane (${\text{C}}_{2}{{\text{H}}}_{6})$	–
Ethylene (${\text{C}}_{2}{{\text{H}}}_{4}$)	Carbon dioxide (${\text{CO}}_{2}$)
Acetylene ($${\text{C}}_{2}{{\text{H}}}_{2})$$

**Table 3:** Types of DGs according to the fault type and material concerned.

Fault type	Flammable	Non-flammable
Overheating of windings	${\text{CO}}$	–
Oil overheating	${\text{CH}}_{4}\text{, }{\text{C}}_{2}{{\text{H}}}_{6}$,${\text{C}}_{2}{{\text{H}}}_{4}$	–
Partial discharge	${\text{H}}_{2}$	–
Arcing	${\text{C}}_{2}{{\text{H}}}_{2}$	–
Minimal temperatures	${\text{CH}}_{4}\text{, }{\text{C}}_{2}{{\text{H}}}_{6}$	–
Excessive temperatures	${\text{C}}_{2}{{\text{H}}}_{4}, {\text{ H}}_{2}$, ${\text{CH}}_{4}\text{, }{\text{C}}_{2}{{\text{H}}}_{6}$	–
All faults	–	${\text{O}}_{2}$, ${\text{N}}_{2}$,${\text{CO}}_{2}$

These approaches are experimental, with assumptions established on relationships between gases discovered through gas analysis^54^. For instance, The Rogers Ratio technique considers the ratios of ${\text{H}}_{2}$, ${\text{CH}}_{4}$, ${\text{C}}_{2}{{\text{H}}}_{6}$, ${\text{C}}_{2}{{\text{H}}}_{4}$, and ${\text{C}}_{2}{{\text{H}}}_{2}$ to create code acknowledging fault analysis. The ratio range, related codes, and related diagnostics for the several code combinations are provided in Table 13. The ratios of the approaches are illustrated as: ***R1:***$\left({\text{CH}}_{4}\text{/}{\text{H}}_{2}\right)$*, ****R2:***$\left({\text{C}}_{2}{{\text{H}}}_{2}\text{/}{\text{C}}_{2}{{\text{H}}}_{4}\right)$*; R3:*$\left({\text{C}}_{2}{{\text{H}}}_{2}\text{/C}{\text{H}}_{4}\right)$*; R4:*$\left({\text{C}}_{2}{{\text{H}}}_{6}\text{/}{\text{C}}_{2}{{\text{H}}}_{2}\right)$*; and R5:*$\left({\text{C}}_{2}{{\text{H}}}_{4}\text{/}{\text{C}}_{2}{{\text{H}}}_{6}\right)$*.*

### CIGRE approach

**Figure 7:**
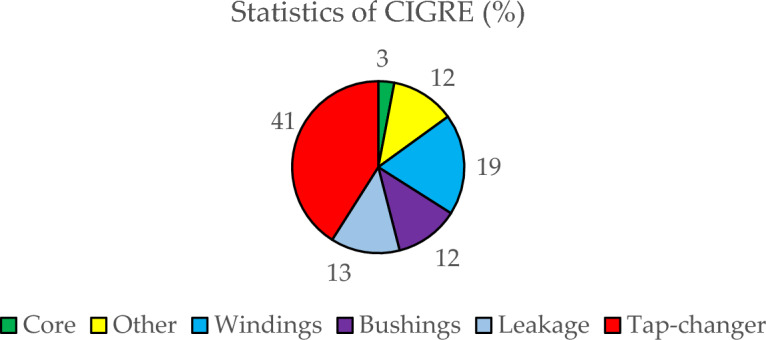
Catastrophe statistics of distinct transformer parts reported by CIGRE^57^.

This approach^55^ explores KG ratios and gas levels. The 5 KG ratios evaluated using this approach are ${\text{C}}_{2}{{\text{H}}}_{2}\text{/}{\text{C}}_{2}{{\text{H}}}_{6}$,${\text{H}}_{2}\text{/C}{\text{H}}_{4}$,${\text{C}}_{2}{{\text{H}}}_{4}\text{/}{\text{C}}_{2}{{\text{H}}}_{6}$,${\text{C}}_{2}{{\text{H}}}_{2}\text{/}{\text{H}}_{2}$, and $\text{CO/C}{\text{O}}_{2}$. A transformer is declared efficient if consecutive deployments of these approaches produce gas ratios and levels that are within permissible thresholds. The incidence of catastrophes in PTs discussed in Ref.^56^ is graphically shown in Fig. 7. The catastrophe statistics of CIGRE consist of approximately 800 catastrophes^57^.

### Doernenburg ratio approach

Reserve integrated safety sensors (RIS2) are required in this approach, and a significant volume of gas is required to demonstrate its application. RIS2 is an essential accessory in the protection of oil transformers. It allows continuous control of the pressure, temperature, oil level, and gas states. The work presented in Ref.^58^ states that gas ratio approaches utilize encoding algorithms that designate certain pairings of codes to specific fault categories for efficient fault identification. The codes are formed by estimating gas level ratios as well as comparing them to predefined values acquired from experience and constantly modified. The author discovered that when a gas composition matches the code for a certain fault, a fault state is identified. In Ref.^59^, the approach was applied to diagnose faults by monitoring gas levels of ${\text{C}}{\text{H}}_{4}\text{/}{\text{H}}_{2}$, ${\text{C}}_{2}{{\text{H}}}_{2}\text{/C}{\text{H}}_{4}$, ${\text{C}}_{2}{{\text{H}}}_{4}\text{/}{\text{C}}_{2}{{\text{H}}}_{6}$ and ${\text{C}}_{2}{{\text{H}}}_{2}\text{/}{\text{C}}_{2}{{\text{H}}}_{4}$. To determine whether there is an actual fault with the unit, the concentration of the gases must initially exceed the acceptable limits, and is adequate formation of each gas for the ratio analysis must be present to be valid^60^. Table 4 demonstrates the KGs as well as their concentration limits.

**Table 4:** Concentration for Doernenburg ratio scheme.

Doernenburg ratio scheme	
KG	Concentration (ppm)
${\text{C}}_{2}{{\text{H}}}_{2}$(acetylene)	35
${\text{C}}_{2}{{\text{H}}}_{6}$(ethane)	65
${\text{C}}_{2}{{\text{H}}}_{4}$(ethylene)	50
${\text{CH}}_{4}$(methane)	120
${\text{CO}}$(carbon monoxide)	350
${\text{H}}_{2}$(hydrogen)	100

### KG approach

This approach^61,62^ monitors the gases emitted from TO upon a failure, which causes the temperature in the transformer to rise. It must be noted that the utility of oil in the transformer provides insulation, and cooling, and helps quench arc. This approach is the most critical and commonly utilized since it presents the earliest signal of an incident. Table 5 demonstrates the diagnostic explanations by using different KG concentrations. The ppm concentration standard value limit detected in PTs according to IEC 60599:2022 is specified in Table 6. The faults in this approach are compared with the gas concentration profile. According to the IEEE standard, KGs are gases produced in OITs that alert to observational fault-type diagnostics, depending on which gases are common or prominent at certain temperature levels^63^. If there is no previous DG data is provided for analysis, hazards in the apparatus can be detected and evaluated using the guidelines indicated in Table 7. Healthy operation is represented by State 1. State 2 signifies that the instrument is possibly malfunctioning, with overall gases exceeding normal concentrations. State 3 implies a high amount of degradation. State 4 indicates that excessive deterioration and continued operation may end in failure or breakdown^64,65^.

**Table 5:** Gas dissolved in oil for analysis.

KG scheme	
Gas detected	Interpretation
${\text{C}}_{2}{{\text{H}}}_{2}$(acetylene)	Electric fault (arc, spark)
${\text{C}}_{2}{{\text{H}}}_{6}$(ethane)	Secondary indicator of thermal fault
${\text{C}}_{2}{{\text{H}}}_{4}$(ethylene)	Thermal fault (overheating local)
${\text{CH}}_{4}$(methane)	Secondary indication of arc or severe excessive heat
${\text{CO}}$(carbon monoxide)	Paper degradation
${\text{CO}}_{2}$(carbon dioxide)	Paper degradation
${\text{H}}_{2}$(hydrogen)	Electromagnetic disposal
${\text{O}}_{2}$(oxygen)	Transformer seal fault

**Table 6:** Limit concentrations of DGs for values observed in the transformer.

Gas	${\text{H}}_{2}$	${\text{CH}}_{4}$	${\text{C}}_{2}{{\text{H}}}_{2}$	${\text{C}}_{2}{{\text{H}}}_{4}$	${\text{C}}_{2}{{\text{H}}}_{6}$	${\text{CO}}$	${\text{CO}}_{2}$
Concentration (ppm)	100	120	1	50	65	350	2500

**Table 7:** Risk assessment in transformers using DG levels (ppm).

States	${\text{H}}_{2}$	${\text{CH}}_{4}$	${\text{C}}_{2}{{\text{H}}}_{2}$	${\text{C}}_{2}{{\text{H}}}_{4}$	${\text{C}}_{2}{{\text{H}}}_{6}$	${\text{CO}}$	${\text{CO}}_{2}$	Total gas
State 1	100	120	1	50	65	350	2500	720
State 2	101–700	121–400	2–9	51–100	66–100	351–570	2500–4000	721–1920
State 3	701–1800	401–1000	10–35	101–200	101–150	571–1400	4001–10,000	1921–4630
State 4	$>$ 1800	$>$ 1000	$>$ 35	$>$ 200	$>$ 150	$>$ 1400	$>$ 10,000	$>$ 4630

### Nomograph approach

**Figure 8:**
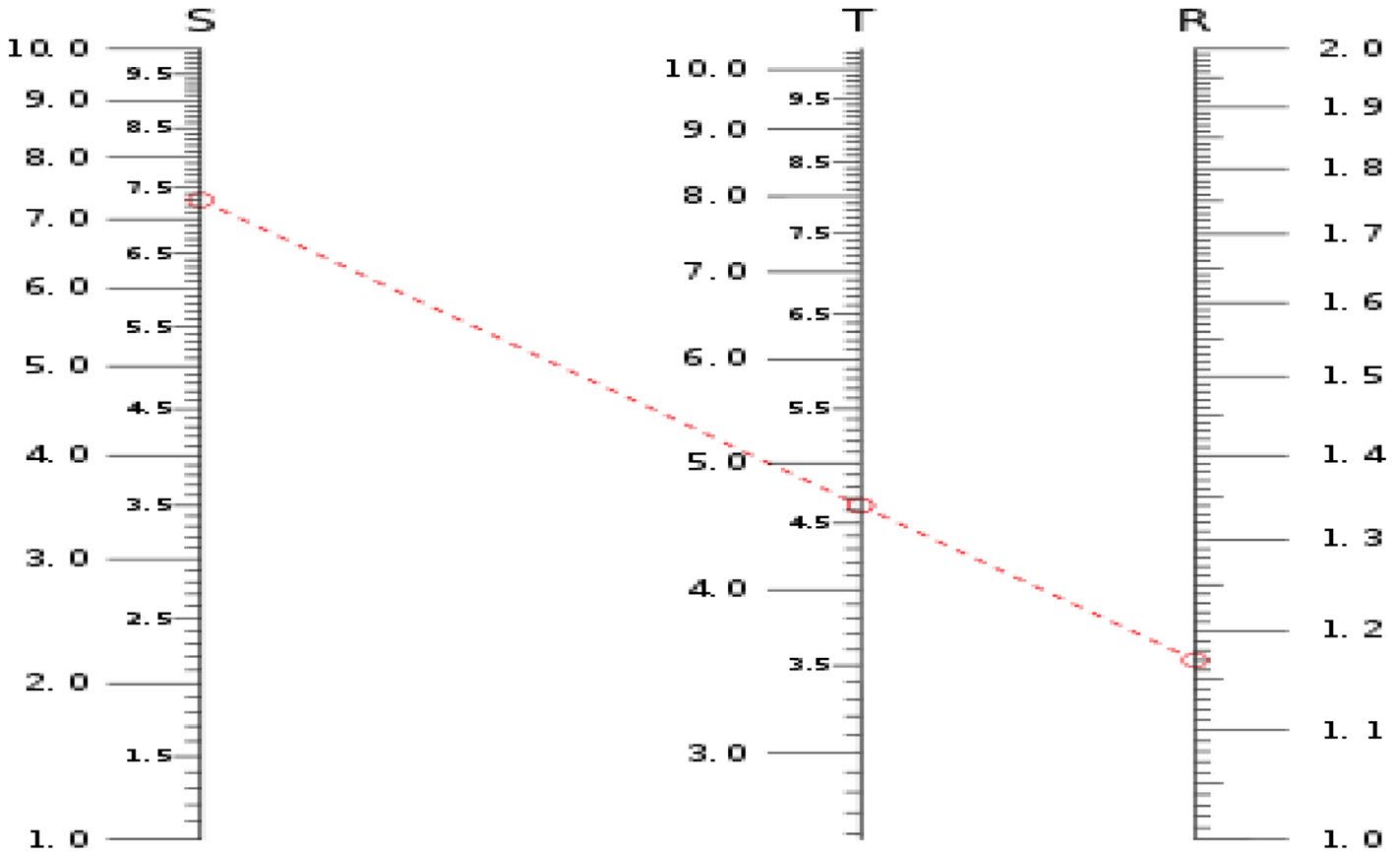
The logarithmic nomograph.

The author^66^ proposed the Nomograph approach to enhance fault analysis precision through the combination of fault gas ratios^67^. It was proposed to supply both a visual demonstration of fault-gas data as well as the resources to understand its importance. The Nomograph is built from upright exponential measurements that depict the amounts of various gases. Using this methodology, straight lines are created between neighboring measurements to link the dots reflecting different gas level values. The gradients of these lines serve as a guide for diagnosing the type of fault. The key (T) across the two axes specifies the fault type for the two axes. The positioning of the line about the intensity scales allows you to determine the degree of the fault. The example to calculate the value of T is shown in Fig. 8.

The formula to calculate the value of *T* is given in (1).

1 $$\text{T } = (\text{1.84S + 4.66})^{0.37}+(\text{1.21R})^{1.333},$$

where S is the pre-test gas, R is the post-test gas and T is the likelihood fault type ratio.

### IEC ratio approach

This approach^68^ analyzes and predicts transformer faults using the first five gases provided in Table 8. The gases are employed to harvest three gas ratios, namely: ${\text{C}}_{2}{{\text{H}}}_{2}\text{/}{\text{C}}_{2}{{\text{H}}}_{4}$, ${\text{CH}}_{4}\text{/}{\text{H}}_{2}$ as well as ${\text{C}}_{2}{{\text{H}}}_{2}\text{/}{\text{C}}_{2}{{\text{H}}}_{6}$. There are two critical considerations to emphasize regarding the approach. Different countries utilize differing ratios, as well as $\left({{\text{the C}}_{2}{\text{H}}}_{2}\text{/}{{\text{C}}_{2}{\text{H}}}_{6}\right)$ ratio usually employed to substitute the $\left({\text{CH}}_{4}\text{/}{\text{H}}_{2}\right)$ ratio. The ratios should be identified mainly when one of the DGs contains a significant concentration and/or a fast-rising rate^69^. Table 9 depicts the IEC standard for describing fault types and provides estimates for the 3 KG ratios using prescribed fault analysis. Whenever key-gas ratios exceed certain limitations, incipient faults in the transformer are to be foreseen^70^.

**Table 8:** Evaluation of DGA data and categorization of fault categories by IEC 60,599.

Fault type	Report	${\text{C}}_{2}{{\text{H}}}_{2}\text{/}{\text{C}}_{2}{{\text{H}}}_{4}$	${\text{CH}}_{4}\text{/}{\text{H}}_{2}$	${\text{C}}_{2}{{\text{H}}}_{2}\text{/}{\text{C}}_{2}{{\text{H}}}_{6}$
PD	Partial discharges	Insignificant value	$<$ 0.1	$<$ 0.2
D1	Low energy discharges	$<$ 1.0	0.1–0.5	$>$ 1.0
D2	High energy discharges	0.6–2.5	0.1–1.0	$>$ 2.0
T1	Thermal faults, T < 300 °C	Insignificant value	Insignificant value	$<$ 1.0
T2	Thermal faults, 300 °C < T < 700 °C	$<$ 1.0	$>$ 1.0	1.0–4.0
T3	Thermal faults, T < 700 °C	$<$ 0.2	$>$ 1.0	$>$ 4.0

**Table 9:** Zone limitations are represented graphically.

Fault type	Fault definition	${\text{CH}}_{4}$	${\text{C}}_{2}{{\text{H}}}_{4}$	${\text{C}}_{2}{{\text{H}}}_{2}$
PD	Partial discharges	98%	–	–
D1	Low energy discharges	–	23%	13%
D2	High energy discharges	–	23–40%	13–29%
T1	Thermal faults	–	20%	4%
T2	Thermal faults	–	20–50%	4%
T3	Thermal faults	–	50%	15%
D + T	Thermal and electrical faults	–	40–50%	4–29%

### Duval triangle approach

**Figure 9:**
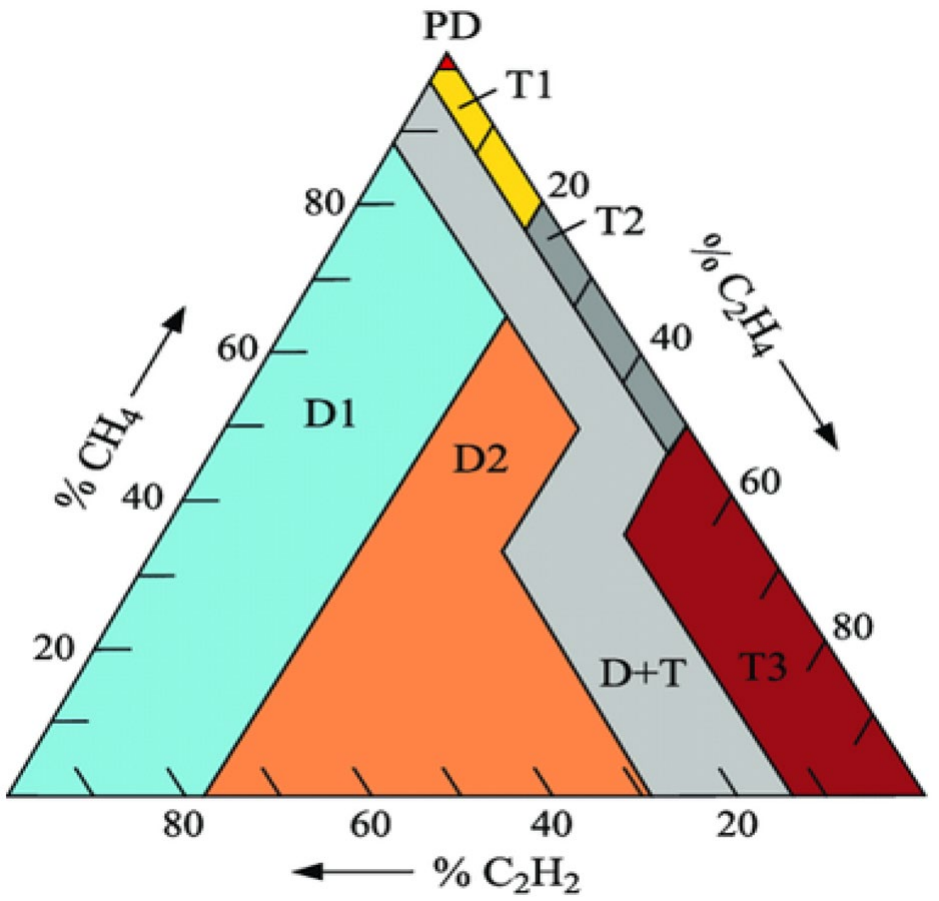
Coordinates and zones of Duval triangle transformer fault diagnosis^79^.

The work in Ref.^71^ analyzes DG data by utilizing a triangle of comparative percentages of ${\text{CH}}_{4}$, ${\text{C}}_{2}{{\text{H}}}_{2}$, and ${\text{C}}_{2}{{\text{H}}}_{4}$. These gases are converted into triangular data to represent the triangle. Even though this approach is regarded as simple to implement, incorrect classifications might occur because no section of the triangle is identified as a sample of typical aging^72,73^. Therefore, before adopting this approach to examine transformers that have been in operation for several years, the acceptable level of DG must be identified. A fault is detected by summing the quantities of the three Duval Triangle gases ${\text{CH}}_{4}$, ${\text{C}}_{2}{{\text{H}}}_{2}$, and ${\text{C}}_{2}{{\text{H}}}_{4}$ as well as extrication the sum by the volume of each gas to get the proportion of each gas overall. The intensities of ${\text{CH}}_{4}\text{, }{\text{C}}_{2}{{\text{H}}}_{4}\text{, as well as }{\text{C}}_{2}{{\text{H}}}_{2}$ are indicated as a proportion of the overall (${\text{CH}}_{4}\text{ + }{\text{C}}_{2}{{\text{H}}}_{4}\text{ + }{\text{C}}_{2}{{\text{H}}}_{2})$ and specify a point ${\text{(\%CH}}_{4}\text{, }{\text{\%C}}_{2}{{\text{H}}}_{4}\text{, and }{\text{\%C}}_{2}{{\text{H}}}_{2})$ in an organized structure denoted in a triangle, which has been sub-divided in separate zones^74^. Each zone is correlated to a specific class of fault. The Duval triangle solely contains areas correlated to fault events; there is no area for normal states. As a result, this approach can only be utilized to classify the kind of fault in the situation of a defective transformer^75,76^. Figure 9 shows the fault type identified in each zone. Internal transformer failures are classified into five kinds based on DL/T 722–2000 and IEC 60599–2022 standards: partial discharge (PD), low energy discharge (D1) and high energy discharge (D2), thermal faults; < 300 °C (T1), thermal fault; 300 °C–700 °C (T2) and a combination of thermal and electrical faults (DT)^77,78^. Figure 9 may be interpreted in a table that shows the fault limitations, which are represented in Table 8.

### Rogers ratio approach

This approach adopts the exact protocol as the Doernenburg Approach, but just three proportions are utilized, and the approach’s validity is not dependent on the gas concentration threshold^71,80^. This approach is proficient since it compares the outcomes of several failure diagnoses with the gas testing of each trial. Nevertheless, certain ratios in this approach are inconsistent with the analytic codes allocated for specific faults^81^. Faults are diagnosed using a basic coding technique based on ratio intervals, as illustrated in Tables 10 and 11^82^. Table 12 shows the proportions for DG in the oil as well as free gases, including the indicated failure detection based on the Rogers Approach. The coding results in 12 distinct forms of transformer malfunctions. Table 13 shows the different types of faults depending on the code.

**Table 10:** Gas ratio code.

Gas ratios	Ratio codes
${{\text{CH}}_{4}\text{/H}}_{2}$	i
${\text{C}}_{2}{{\text{H}}}_{6}{\text{/CH}}_{4}$	j
${\text{C}}_{2}{{\text{H}}}_{4}{\text{/C}}_{2}{{\text{H}}}_{6}$	k
${\text{C}}_{2}{{\text{H}}}_{2}\text{/}{\text{C}}_{2}{{\text{H}}}_{4}$	l

**Table 11:** Coding of Roger’s ratios.

Ratio code	Interval	Code
i	$\le$ 0.1	5
	$>$ 0.1, $<$ 1.0	0
	$\ge$ 1.0,$<$ 3.0	1
	$\ge$ 3.0	2
j	$<$ 1.0	0
	$\ge$ 1.0,$<$ 3.0	1
k	$<$ 1.0	0
	$\ge$ 1.0,$<$ 3.0	1
	$\ge$ 3.0	2
l	$<$ 0.5	0
	$\ge$ 0.5,$<$ 3.0	1
	$\ge$ 3.0	2

**Table 12:** Categorization based on Roger’s ratio code.

i	j	k	l	Analysis
0	0	0	0	Common degradation
5	0	0	0	Partial discharge
1–2	0	0	0	Slight overheating < 150 °C
1–2	l	0	0	High-temperature 150 °C–200 °C
0	1	0	0	High-temperature 200 °C–300 °C
0	0	1	0	General conductor overheating
1	0	1	0	Winding flowing currents
1	0	2	0	Core and tank overheating links
0	0	0	1	Flashover without power follow-through
0	0	1–2	1–2	Arc with energy follow-through
0	0	2	2	Constant flashing to free potential
5	0	0	1–2	Partial discharge with stalking (note CO)

**Table 13:** Correlations for DG in oil, free gas, and catastrophe analysis are recommended by the approach of Rogers.

Event	R2,${\text{C}}_{2}{{\text{H}}}_{2}\text{/}{\text{C}}_{2}{{\text{H}}}_{4}$	R1,${\text{CH}}_{4}\text{/}{\text{H}}_{2}$	R5,${\text{C}}_{2}{{\text{H}}}_{4}\text{/}{\text{C}}_{2}{{\text{H}}}_{6}$	Catastrophe analysis recommended
0	$<$ 0.1	$>$ 1.0$, <$ 0.1	$<$ 0.1	Healthy unit
1	$<$ 0.1	$>$ 1.0	$<$ 0.1	Partial discharge
2	1.0–3.0	0.1–1.0	$>$ 3.0	High energy discharge
3	$<$ 0.1	$>$ 1.0, $<$ 0.1	1.0–3.0	Low-temperature thermal failure
4	$<$ 0.1	$>$ 1.0	1.0–3.0	Thermal collapse $<$ 700 °C
5	$<$ 0.1	$>$ 1.0	$>$ 3.0	Thermal collapse $>$ 700 °C

## Applicable works

The seven (7) DGA approaches provided in Section “Review of existing DGA approaches” are performed to monitor as well as synthesize the importance of gases existing in OITs. Concerning the faults of the above-mentioned conventional schemes, artificial intelligence (AI) schemes of PT fault analysis have attracted substantial consideration due to their superior flexibility and influential fault analysis presentation (e.g. expert system (EPS)^83^, fuzzy theory^84^, SVM^85^, extreme learning machine (ELM)^86^, as well as ANN^87^). EPS remains a clever AI setup scheme linked with skilled knowledge, which can analyze faults more thoroughly, precisely, and instantly.

For instance, in Refs.^49,88^, the author built an EPS for PT insulation fault analysis, which undertook DGA as the normal factor. The analysis results demonstrated that the suggested EPS can thoroughly examine the insulation state of a unit and detect the type of fault accurately. In Ref.^89^, the authors reported an instinctive fuzzy EPS to analyze PT faults, in such a manner that the approximation of KG ratio in the TO can be easier. The fuzzy concept mostly analyzes the interrelations amongst fuzzy matters, so it can handle these matters appropriately with fuzziness and ambiguity.

Also, the authors in Ref.^90^ applied fuzzy logic linked with evaporated gas of crystal oil for PT fault analysis. Experimental outcomes proved that the extremely useful fault analysis scheme was to syndicate outputs from several DGA approaches as well as to combine them into a complete assessment.

The authors in Ref.^91^ found the smart analysis logic centered on principal component analysis (PCA) as well as an adaptable evaluation scheme under fuzzy logic facilitates to forecasting initial fault analysis of PTs. SVM is an AI system based on the numerical learning hypothesis which holds impressive benefits in non-linear complications. The author^92^ examined a new extension technique in which an SVM was utilized to examine the PT’s faults and to elect the extremely applicable gas signature among the DGA conventional approaches and a new extension technique. The examination outcomes showed that the new extension technique as well as the SVM scheme can notably enhance the analysis precisions for PT fault categorization.

The authors in Ref.^93^ suggested an improved prototype merging SVM with a genetic algorithm (SVMG) to analyze PT faults. The trial outcomes revealed that the SVMG technique can accomplish better ranking analytical precision compared to the IEC three ratios, typical SVM classifier, as well as ANN. ELM is a developing learning procedure that has been initiated for transformer fault analysis in current years. In Ref.^3^, the author employed ELM mixed with PCA to categorize the initial faults of PTs and assessed its execution with fuzzy logic as well as ANN. The evaluated outcomes demonstrated that ELM could supply decent analysis findings. Again in Ref.^51^, the author proposed an integrated particle swarm optimization (PSO) as well as an ELM technique to analyze PT faults.

Despite that, these analysis techniques examined earlier retain their fundamental disadvantages as follows: (i) For EPS, a complete understanding root is a vital feature to guarantee the precision of analysis. Nevertheless, it is challenging to acquire a comprehensive knowledge base. Moreover, the EPS produces inadequate understanding capability; (ii) Fuzzy theory is challenging to establish a suitable link equation linking the input and output parameters^94^; (iii) SVM is a double-categorization procedure, that causes difficulty in terms of constructing an acquiring mechanism, choosing kernel features, and establishing variables in dual-classification challenges. Consequently, SVM has the inherent deficiency of low categorization effectiveness^95,96^; (iv) The execution of ELM is not balanced given that its concealed layer variable is casually selected. Compared to the fault analysis techniques discussed in Section “Review of existing DGA approaches”, the neural network has an extra general function in fault analysis of PTs due to its lack of sophistication, solid nonlinear-fitting capability, and high accuracy. For instance, the authors in Ref.^97^ used a neural-fuzzy network to determine the initial faults in PTs, as well as to execute and examine the anticipated procedure using simulation trials. In Ref.^98^ the author published validated research for selecting the best multi-layer perceptron (MLP) neural network simulation through comparisons of two output data kinds and three concealed layer categories. According to the trial findings, MLP neural network ratio amalgamation simplifies more accurately compared to different MLP neural network simulations.

In Ref.^99^, the author presented an ML-based scheme for PT fault analysis based on DGA, a bat algorithm (BA), as well as improving the probabilistic neural network (PNN). Investigation revealed that the recommended ANN-based approach was detected more precisely when compared to the Rogers ratios technique when a DGA technique centered on ANN was applied. The back propagation neural networks (BPNN) model is a highly common one amongst several neural network processes and it is being broadly utilized in various grounds of fault analysis. In particular, power electronic systems^100^, transformers^101^, batteries^102,103^, photovoltaic systems (PV)^104,105^, etc. be that as it may, the BPNN model still has several underlying deficiencies, for instance, dull merging speed and over-fitting difficulty^106,107^. Fortunately, a significant compilation of optimization processes has been established to optimize the BPNN version, such as Genetic algorithm (GA)^108–111^, Means end analysis (MEA)^112^, Particle Swarm Optimization (PSO)^113,114^, Simulated Annealing (SA)^115^, BA^116,117^, etc. Adaptive systems, including GA and MEA, are among these techniques and are currently utilized as optimizing approaches aiming for the perfect weights and limitations of ANNs.

## Proposed approach

In this work, a multi-classification model that is based on ML algorithms is presented to have an intelligible, precise, and clear understanding of DGA. Transformers are pivotal equipment in the transmission and distribution of electrical power. The failure of a particular unit during service may interrupt a massive number of consumers and disturb commercial activities in that area. Therefore, several monitoring techniques are proposed to ensure that the unit maintains an adequate level of functionality in addition to an extended useful lifespan. DGA is a technique commonly employed for monitoring the state of OITs. The understanding of DGA samples is however unsatisfactory from the perspective of evaluating incipient faults and relies mainly on the proficiency of test engineers.

**Figure 10:**
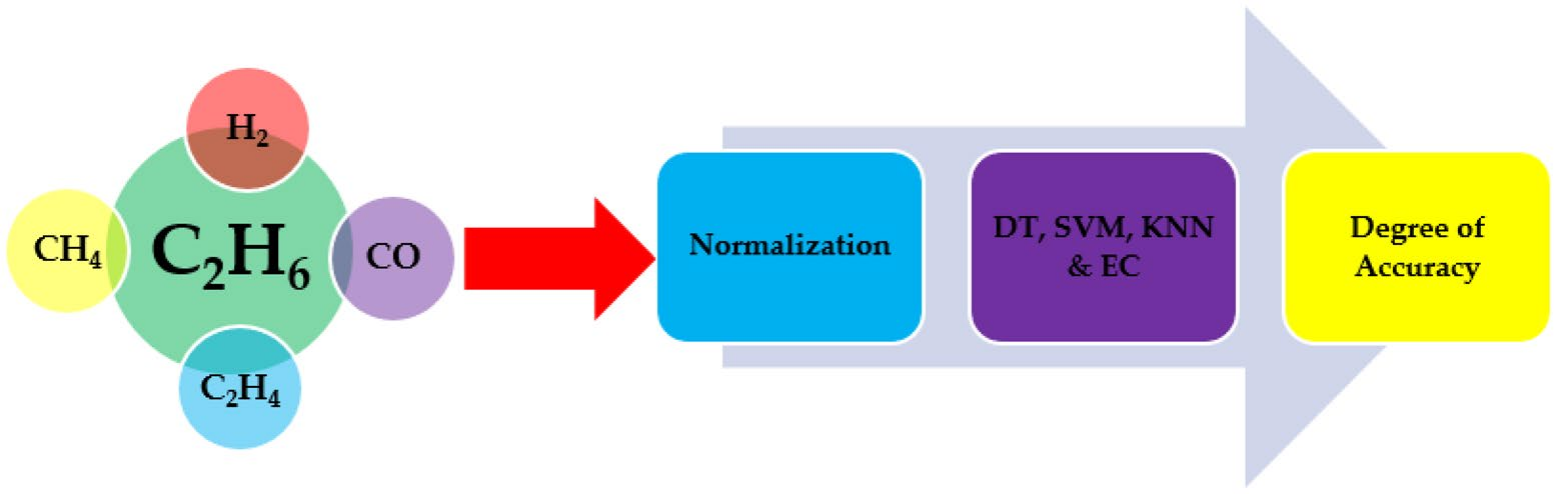
Research flowchart for MC model.

The proposed model is utilized to investigate as well as assess the state and suitable gas name subscription of 138 TO samples that revealed different stray gassing characteristics in various South African substations. This is achieved by employing four ML classifiers. The advantages of the selected classifiers are discussed in Section “Introduction” of this study. The primary objective of this study requires the development of an ML-based health index (HI) model. It is suggested that HI be used to forecast the predicted output parameter, which is conceptually connected to the input characteristics, centered on the crucial assessment^4^. SVM can generate fresh information and categorize non-linear problems. KNN, in comparison, serves as a reliable, trained ML classifier that may be used to address categorization and prediction issues. Its primary drawback is the fact that it gets much more sluggish as the amount of data being used grows. During the normalizing step, the normalized ratio of every gas in every data collection sample is introduced. Figure 10 shows a flowchart that depicts an outline of the planned research.

### Dataset preparation

**Figure 11:**
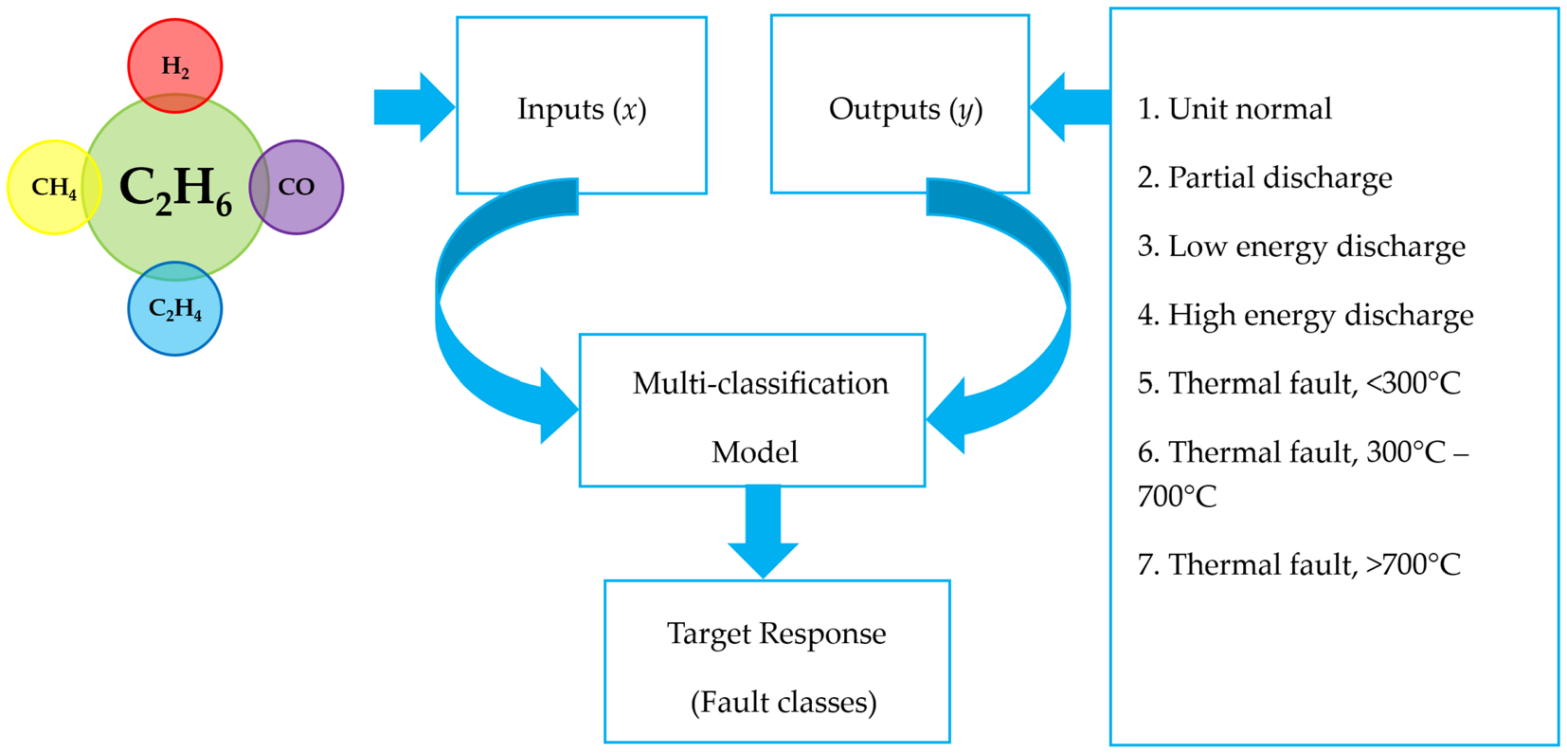
Function block diagram on the proposed model.

Relating to the 138 oil samples, 83 oil samples are utilized as training data, 25 oil samples as testing data, and the remaining 30 oil samples for validation purposes. The DT, SVM, KNN, and EC classifiers serve as a parameter to the HI model to forecast a trait. As a result, the presented HI computation technique will be considerably less costly. HI prognosis classifications are utilized as feature inputs with the transformer, and monitored modeling is applied. The developed HI framework must be solidly verified before it can be employed in practical applications. The dataset is loaded and distributed into the feature inputs (*x*) as well as feature outputs (*y*). The feature inputs are gases: $\left({\text{H}}_{2}\right)$, $\left({\text{CH}}_{4}\right)$, $\left({\text{C}}_{2}{{\text{H}}}_{4}\right)$, $\left({\text{C}}_{2}{{\text{H}}}_{6}\right)$, and $\left({\text{CO}}\right)$ level in ppm, and feature outputs are faults category. In this present research, 83 oil samples are classified as training, 25 oil samples as evaluation, and the final 30 oil samples as verification. In Fig. 11, the function block diagram on the proposed model is illustrated for the diagnosis of various incipient transformer faults.

The proposed model generates a system response based on the feature inputs (*x*) as well as targeted feature outputs (*y*) absorbed into the network. The construction of a multi-classification model includes the identification of the best-performing system training techniques and parameters. In this present research, parameters are developed implicitly by considering efficiency and network reliability. The diagnostic gas contents acquired by DGA serve as the experimental base for diagnosis. The content data reflects the transformer conditions. These diagnostic gases include $\left({\text{H}}_{2}\right)$, $\left({\text{CH}}_{4}\right)$, $\left({\text{C}}_{2}{{\text{H}}}_{4}\right)$, $\left({\text{C}}_{2}{{\text{H}}}_{6}\right)$, and $\left({\text{CO}}\right)$. To increase the effect of classification, the contents of these diagnostic gases are pre-processed using a specific data processing method, and seven features for fault diagnosis are extracted for the proposed model. From the successful implementation of the model, it can be concluded that the model has some potential advantages, which are as follows:

- It is a highly regularized strategy that is suitable for ill-posed issues.
- It offers a unique approach and has a high training curve/speed.

### Experimental setup

The training databank of 138 TO samples is used to develop the proposed model. A *k*-fold cross-validation method is employed in the development of the model. The *k*-fold cross-validation method is employed to assess the model's competence with new data. The procedure has a single parameter called *k* that refers to the number of groups that a given data sample is to be split into. In the setup, a 30-fold cross-validation approach is employed to execute the experiment. As a result, during the 30-fold validation process, the operation is performed numerous times with varied partitions of the data findings into 30 parts. When a specific value for *k* is selected, it may be used in place of *k* in the reference to the model, such as *k* = 30 becoming 30-fold cross-validation. It implies that the model will be evaluated several times to increase confidence in the model design. This eliminates the concept of training the model only once and not knowing if the positive result is due to luck or not. Cross-validation is a strategy for testing ML models that involves training numerous ML models on subsets of the given input data and then evaluating them on the corresponding subset. Cross-validation can be used to detect overfitting, or the failure to generalize a pattern. Performing 30-fold cross-validation generates 30 models, 30 data sources to train the models, 30 data sources to evaluate the models and 30 evaluations, one for each model.

In the present study, to evaluate the efficiency, several classifiers were put to work: (i) DT, (ii) SVM, (iii) KNN, and (iv) EC. Consequently, before the construction of the ML model, the settings of the proposed classifiers need to be established. Table 14 summarizes the ML setting configuration. The forward selection feature of stepwise regression^118^ is used in the setup. Each term is either removed or included as a feature input vector according to the *p*-value of the present or newly entered data inputs. The *p*-value determines the likelihood it is to obtain a certain result when the null-hypothesis is assumed to be true. The null-hypothesis is the argument in scientific study, that no relationship exists between two sets of data or variables being trained/tested. The null-hypothesis states that any empirically observed difference is due only to chance and that no fundamental causal relationship exists, thus the word "null"^119^. As a result, if the null-hypothesis is considered to be true, the *p*-value estimates how odd the tested sample is. The likelihood of a null-hypothesis experiment is denoted as a *p*-value using *the α* parameter for term addition as well as the *β* parameter^120^ for term deletion. The *α* and *β* parameters are the threshold values against which *p*-values are measured. It demonstrates how significant the observed results must be for a significance test to reject the null-hypothesis. Every data entry point indicates a different form of gas collected from DGA. The *p*-value following a stepwise regression analysis was used to choose the input data. The benefit of the *p*-value is that its parameters can be experimentally modified to achieve the best results.

**Table 14:** Summary of MC model configuration.

No	Classifier	Parameter
1	DT	Highest no. of splits: 121, Splitting criteria: Towing rule
2	SVM	Regularization = 12 norm, Box restriction setting: 896.5514 Kernel operation: Cubic, Loss = Square hinge
3	KNN	No. of neighbour = 30, Distance = Minkowski
4	EC	Ensemble scheme: AdaBoost, No. of trainees: 138, Training ratio: 0.8995, Highest no. of split up: 30

### Training and testing of the ML models

As the input characteristics are supplied through the transformer HI estimation, supervisory training is adopted. 83 of the oil samples are classified as training, 25 as evaluation, and the final 30 oil samples as verification. To accommodate the narrow distribution of data, an evaluation threshold of 30 was utilized, particularly for the "Very Poor" data. In Ref.^121^, even though 83 of the transformer HI classifications are chosen to be utilized for training along with 25 for assessment, minimal transformer HI classifications are evaluated. As a result, obtaining more accurate data distribution is advantageous. Furthermore, a cross-validation approach is adopted for detecting overfitting or underfitting. After the model has been cross-validated, the settings can be adjusted for the next model if it does not meet the required standards. In light of this, a 30-fold cross-validation approach is carried out in a manner comparable to selection in Ref.^122^. The 30-fold validating approach is carried out repeatedly using different 30-part splits within the test findings. Furthermore, in this work, a comparison analysis is also conducted against the conventional DGA approaches to certify the proposed model.

### Classification accuracy

The proposed model is tested using 30-testing datasets. The precision of the classification indicates how frequently a classification algorithm is accurate. The formula for the *Sfn* is given in (2).

2 $${\text{S}}_{\text{fn }}= \text{ } \frac{{\text{P}}_{\text{fn}}}{\text{Number of cases of fn}} \, \times \text{ 100,}$$

where ${\text{S}}_{\text{fn}}$ is the proportion of valid prognosis of a certain fault type$, {\text{fn}}$, and P is the valid prognosis^11^.

Consistency (*C*) indicates the precision of the model in each dataset. This gives an enhanced metric of incorrectly categorized occurrences. The equation for the C is provided in (3).

3 $$\text{C = }\frac{{\sum }_{1}^{\text{fn}}{{\text{S}}}_{\text{fn}}}{\text{Number of fault types}}.$$

$${\text{f}}_{\text{n}}\text{ = type fault code (n=1,2,3,4,5)}$$.

The precision (*A*) of the classifiers is determined by their valid prognosis (*P*) in identifying distinct faults. The precision of (*A*) is calculated as illustrated in (4).

4 $$\text{\%A =}\frac{{\text{T}}_{\text{sp}}}{{\text{T}}_{\text{tc}}} \, \times \text{ 100}$$

where ${\text{T}}_{\text{sp}}$ is the total number of correct predictions and ${\text{T}}_{\text{tc}}$ is the cumulative number of events^76,93,100,105^.

## Materials and protocols

Transformers help diversified settings that make them liable to broad failures whose outcomes are extended occurrences of power outages and disrupted commercial activities. In contrast to an overhead power line that is painless to overhaul, transformers are factory-sealed, denying technicians on-site the ability to inspect their active-part assemblies. The inception of faults of transformers during operation generates considerable hammering of revenues to power utility owners in addition to the excessive damages or replacement expenditures and the probability of an explosion. DGA is the only available scheme that provides the means to feasibly identify incipient transformer faults. Though the estimation precision of DGA schemes remains reasonably eminent, the schemes employed to understand DGA samples remain dependent on the proficiency of test engineers as opposed to precise interpretation. This work consequently sought to introduce and carry out a novel multi-classification ML-based DGA interpretation scheme that explains DGA samples strictly concerning multinomial data sets.

The oil samples used in this research came from several South African power stations. The databank contains five flammable gases initiated from distinct oil specimens collected from transformers in service. Classification Learner App in MATLAB/Simulink was employed to train the gas concentrations of 138 oil samples. The proposed MATLAB model is valid since the coding tool, MATLAB Simulink as well as the toolbox are widely utilized in the research and engineering field. The Classification Learner tool is a highly efficient platform that allows you to engage with data, choose features, establish cross-validation methods, train models, and evaluate outcomes^13,20,90^. It is used for routine activities such as:

- Data import and cross-validation scheme configuration.
- Data exploration as well as feature selection.
- Model training employing different classification tools.
- Model comparison and evaluation.

Researchers can develop and verify classification models by applying different techniques through the use of the Classification Learner app. Analyze the validating flaws of the simulations post-training, then select the most accurate model based on the outcomes. From the data, 83 oil samples are utilized as training data, 25 oil samples as testing data, and the remaining 30 oil samples for validation purposes. Eight classes of faults were detected from the oil data samples with a supplementary label designating a normal gas concentration. In response to 138 oil samples investigated, there is a total of 7 types of faults present as indicated in Table 15. Five gas proportions are assessed by adopting the IEC 60599:2022 guideline proposal considering the six flammable gases extracted in the different oil samples^21^.

**Table 15:** Classification of transformer faults.

Type	Fault
PD	Partial discharge
D1	Low energy discharge
D2	High energy discharge
NF	No-Fault
T1	Thermal fault < 300 °C
T2	Thermal fault, 300˚C < T < 700 °C
T3	Thermal fault, > 700 °C
D + T	–

**Figure 12:**
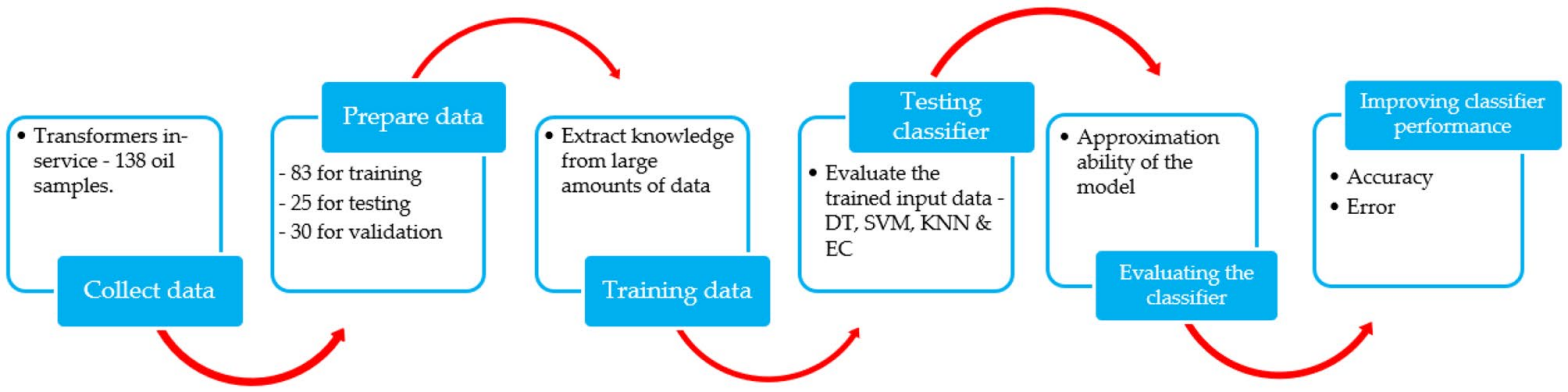
ML workflow.

**Figure 13:**
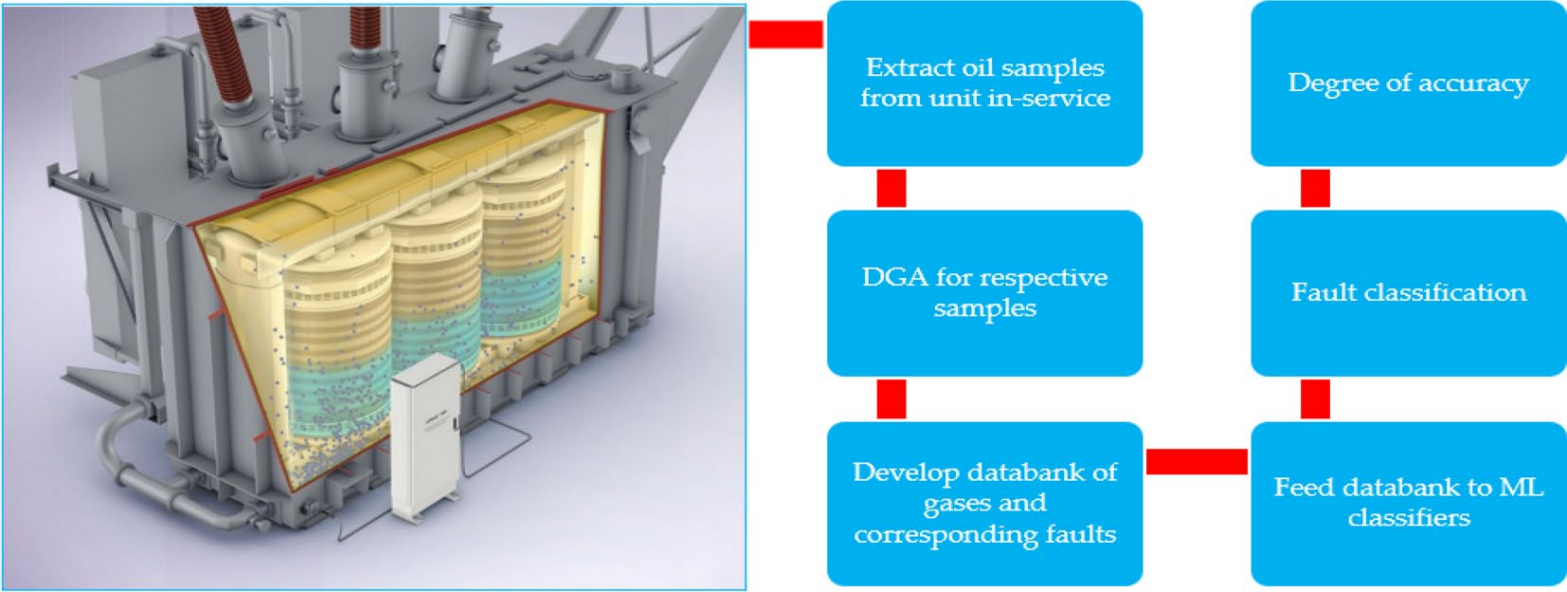
TO evaluation phases.

The objective of the preliminary exercise was to check the oil data samples so that faults may be detected and diagnosed. Following data pre-processing, DGA data clarification began, and a model was established utilizing the stages shown in Fig. 12. The authors provided Fig. 13, which depicts the different phases, to broaden the reader's perception.

## Results

Classification Learner App in MATLAB/Simulink was utilized to train the gas concentrations of 138 oil samples extracted from various South African plants. ML classifiers in the Classification Learner App were selected and a 30-fold cross-validation was used to set the training and testing data for the model. Based on the total dataset, 83 oil samples are utilized as training data, 25 oil samples as testing data, and the remaining 30 oil samples for validation purposes. Cross-validation of 30-folds was selected, which in other words implies that the training and testing operations were repeated 30 times. Stepwise regression was used to choose the gases from the DGA that had the most significant feature for identifying transformer faults from the input (*x*) and output (*y*) data. Table 16 demonstrates the results of stepwise regression. The samples utilized to train as well as test the model have comparable traits. The *p*-value examines the null-hypothesis, and it possesses a likelihood of zero. An indicator with a small *p*-value, such as ${\text{CO}}$, which has a value of 1.0214 × 10–34, is a good contributor to the model in terms of the specified characteristics. A small *p*-value for the gas indicates that the DGA data for that specific gas has a higher connection with the transformer fault type. The standard error is crucial for establishing the robustness of the connection between the predictive model and the reaction variable. Furthermore, standard error provides accessibility to the credibility of the* p*-values since it shows the numerical range that the measured numbers deviate from the prediction line. As demonstrated in Table 16, a smaller standard error correlates to a faster reaction since the model developed provides measurements of the reaction variable, which is the fault type closest to the aptness line.

**Table 16:** Findings of feature extraction utilizing stepwise regression^118^.

Type of gas	Training data			Testing data		
	p-value	Regression coefficient (× 10^–3^)	Standard error (× 10^–3^)	p-value	Regression coefficient (× 10^–3^)	Standard error (× 10^–3^)
${\text{C}}_{2}{{\text{H}}}_{6}$	0.0398	− 0.0156	0.007424	0.0378	− 0.0156	0.007421
${\text{C}}_{2}{{\text{H}}}_{4}$	0.9540	− 0.0006	0.009493	0.9326	− 0.0009	0.009492
${\text{CH}}_{4}$	0.1683	0.0162	0.001128	0.1768	0.0016	0.001506
${\text{CO}}$	1.0214 × 10^–34^	0.0073	0.000359	1.0978 × 10^–34^	0.0072	0.000359
${\text{H}}_{2}$	5.6277 × 10^–21^	0.1694	0.014008	5.3724 × 10^–21^	0.1685	0.013928

The transformer states are classified into four categories: A, B, C, and D, with A signifying excellent state, B signifying fair state, C indicating that servicing is needed, and D signifying a detrimental or failing unit. This is shown in Table 17. The interpretations and constraints offered by national standards such as IEEE, ICE, and Eskom standards are used to identify these categories.

**Table 17:** Transformer categories.

Category	Percentage (%)	Transformer state
A	85–100	Excellent
B	70–84	Fair
C	50–69	Service
D	30–49	Detrimental/failed

**Figure 14:**
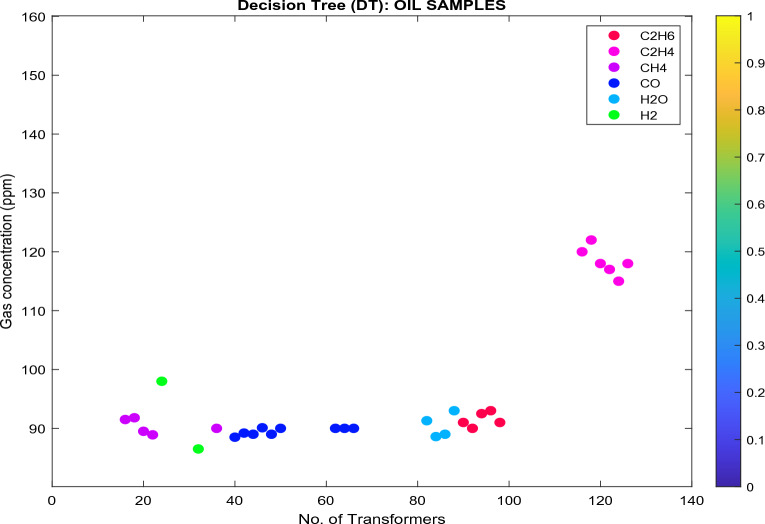
Analyzed databank using DT classifier.

**Figure 15:**
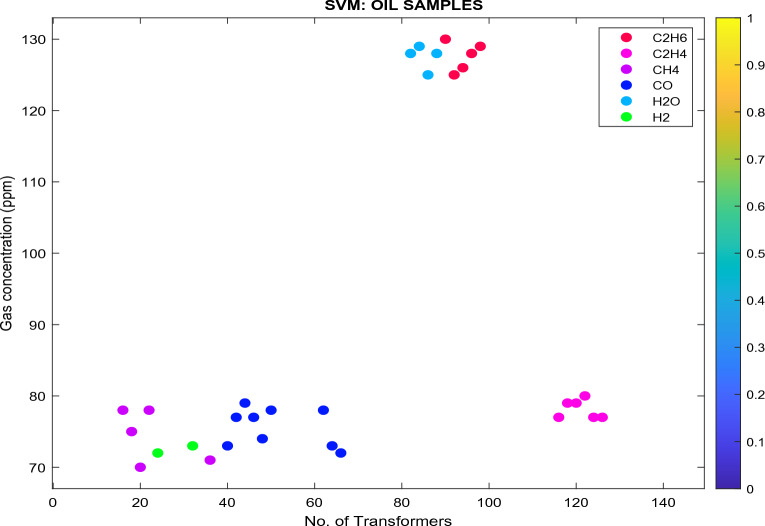
Analyzed databank using SVM classifier.

**Figure 16:**
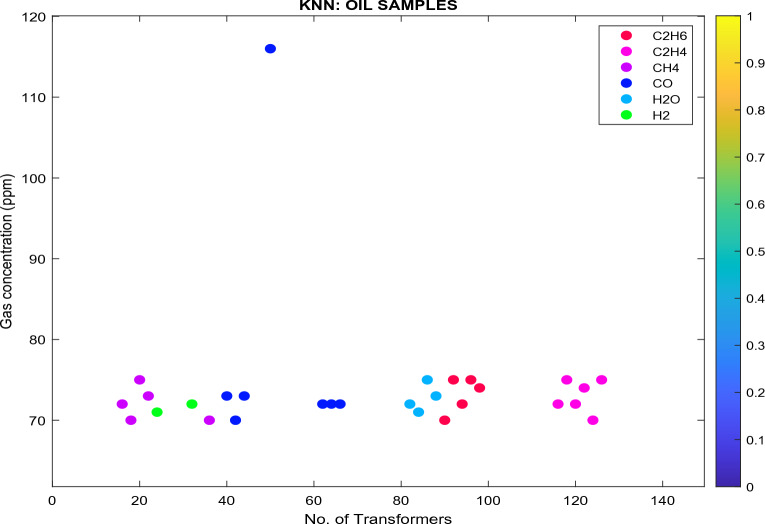
Analyzed databank using KNN classifier.

**Figure 17:**
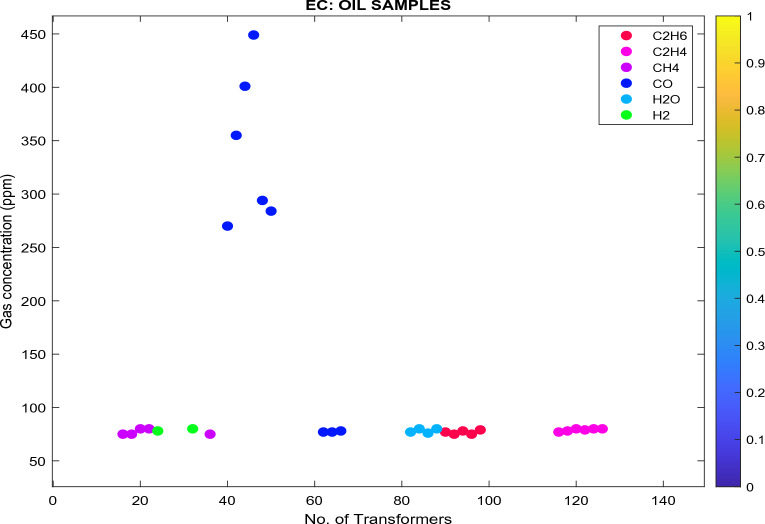
Analyzed databank using EC classifier.

Figures 14, 15, 16 and 17 show the results of the four classifiers employed to analyze and evaluate the state and suitable gas name subscription of 138 TO samples that revealed different stray gassing characteristics in various South African substations. These gases: $\left({\text{H}}_{2}\right)$, $\left({\text{CH}}_{4}\right)$, $\left({\text{C}}_{2}{{\text{H}}}_{2}\right)$, $\left({\text{C}}_{2}{{\text{H}}}_{4}\right)$, and $\left({\text{CO}}\right)$ concentration were detected in 31 transformers. These were based on transformers that were significantly affected by faults. The same dataset was used to train the classifiers, and it was performed to determine how well each classifier performs when underfitting or overfitting occurs. This occurs when the model cannot determine a meaningful relationship between the input (*x*) and output (*y*) data. Underfit models are more probable if they have not been trained for the proper amount of time on a large number of data points.

Therefore, the following observations were made, and suggestions were made based on the findings and consultation with the IEC 60599:2022 and Eskom standard:

In Fig. 14:

- Transformers (in pink) are classified as state 2 since the ${\text{C}}_{2}{{\text{H}}}_{4}$ concentration is between 101 and 700 ppm, as specified in Table 7. These transformers are classified as Class C, as specified in Table 17. The recommendations are: (i) exercise caution; (ii) analyze for individual gases to find cause; (iii) determine load dependence.
- Transformers (in red) are classified as state 2 since the ${\text{C}}_{2}{{\text{H}}}_{6}$ concentration is between 66 and 100 ppm. These transformers are classified as Class B. The recommendations are: (i) exercise caution; (ii) analyze for individual gases to find cause; (iii) determine load dependence.
- Transformers (in purple) are classified as state 4 since the ${\text{CH}}_{4}$ concentration is < 120 ppm. These transformers are classified as Class A. The recommendation is to: (i) No action is required.
- The moisture is 25 ppm (light blue), which is greater than 15 ppm (Eskom Specification—Ref: 240-75661431.). These transformers are classified as Class C. The recommendation is to (i) TO needs refinement/service to enhance the oil state and resampling.
- Transformers (dark blue) are classified as state 1 since the ${\text{CO}}$ concentration is < 350 ppm. These transformers are classified as Class A. The recommendations are: (i) No action is required.
- Transformers (in green) are classified as state 1 since the ${\text{H}}_{2}$ concentration is < 100 ppm. These transformers are classified as Class A. The recommendations are: No action is required.

In Fig. 15:

- Transformers (in pink) are classified as state 2 since the ${\text{C}}_{2}{{\text{H}}}_{4}$ concentration is between 51 and 100 ppm, as specified in Table 7. These transformers are classified as Class B, as specified in Table 17. The recommendations are: (i) exercise caution; (ii) analyze for individual gases to find cause; (iii) determine load dependence.
- Transformers (in red) are classified as state 3 since the ${\text{C}}_{2}{{\text{H}}}_{6}$ concentration is between 101 and 150 ppm. These transformers are classified as Class C. The recommendations are: (i) plan service/maintenance; (ii) analyze for individual gases to find cause; (iii) remove if possible.
- Transformers (in purple) are classified as state 1 since the ${\text{CH}}_{4}$ concentration is < 120 ppm. These transformers are classified as Class A. The recommendation is to: (i) No action is required.
- The moisture is 25 ppm (light blue), which is greater than 15 ppm (Eskom Specification—Ref: 240-75661431.). The recommendation is to (i) TO needs refinement to enhance the oil state and resampling.
- Transformers (dark blue) are classified as state 1 since the ${\text{CO}}$ concentration is < 350 ppm. These transformers are classified as Class A. The recommendations are: (i) No action is required.
- Transformers (in green) are classified as state 1 since the ${\text{H}}_{2}$ concentration is < 100 ppm. These transformers are classified as Class A. The recommendations are: No action is required.

In Fig. 16:

- Transformers (in pink) are classified as state 2 since the ${\text{C}}_{2}{{\text{H}}}_{4}$ concentration is between 51 and 100 ppm, as specified in Table 7. These transformers are classified as Class B, as specified in Table 17. The recommendations are: (i) exercise caution; (ii) analyze for individual gases to find cause; (iii) determine load dependence.
- Transformers (in red) are classified as state 2 since the ${\text{C}}_{2}{{\text{H}}}_{6}$ concentration is between 66 and 100 ppm. These transformers are classified as Class B. The recommendations are: (i) exercise caution; (ii) analyze for individual gases to find cause; (iii) determine load dependence.
- Transformers (in purple) are classified as state 1 since the ${\text{CH}}_{4}$ concentration is < 120 ppm. These transformers are classified as Class A. The recommendation is to: (i) No action is required.
- The moisture is 25 ppm (light blue), which is greater than 15 ppm (Eskom Specification—Ref: 240-75661431.). The recommendation is to (i) TO needs refinement to enhance the oil state and resampling.
- Transformers (dark blue) are classified as state 1 since the ${\text{CO}}$ concentration is < 350 ppm. These transformers are classified as Class A. The recommendations are: (i) No action is required.
- Transformers (in green) are classified as state 1 since the ${\text{H}}_{2}$ concentration is < 100 ppm. These transformers are classified as Class A. The recommendations are: No action is required.

In Fig. 17:

- Transformers (in pink) are classified as state 2 since the ${\text{C}}_{2}{{\text{H}}}_{4}$ concentration is between 51 and 100 ppm, as specified in Table 7. These transformers are classified as Class B, as specified in Table 17. The recommendations are: (i) exercise caution; (ii) analyze for individual gases to find cause; (iii) determine load dependence.
- Transformers (in red) are classified as state 2 since the ${\text{C}}_{2}{{\text{H}}}_{6}$ concentration is between 66 and 100 ppm. These transformers are classified as Class B. The recommendations are: (i) exercise caution; (ii) analyze for individual gases to find cause; (iii) determine load dependence.
- Transformers (in purple) are classified as state 1 since the ${\text{CH}}_{4}$ concentration is < 120 ppm. These transformers are classified as Class A. The recommendation is to: (i) No action is required.
- The moisture is 25 ppm (light blue), which is greater than 15 ppm (Eskom Specification—Ref: 240-75661431). The recommendation is to i) TO needs refinement to enhance the oil state and resampling.
- 3 × Transformers (dark blue) are classified as state 1 since the ${\text{CO}}$ concentration is < 350 ppm. These transformers are classified as Class A. The recommendations are: (i) No action is required. 3 × Transformers (dark blue) are classified as state 2 since the ${\text{C}}{\text{O}}$ concentration is between 351 and 570 ppm. These transformers are classified as Class B. The recommendations are: (i) No action is required.
- Transformers (in green) are classified as state 1 since the ${\text{H}}_{2}$ concentration is < 100 ppm. These transformers are classified as Class A. The recommendations are: No action is required.

**Figure 18:**
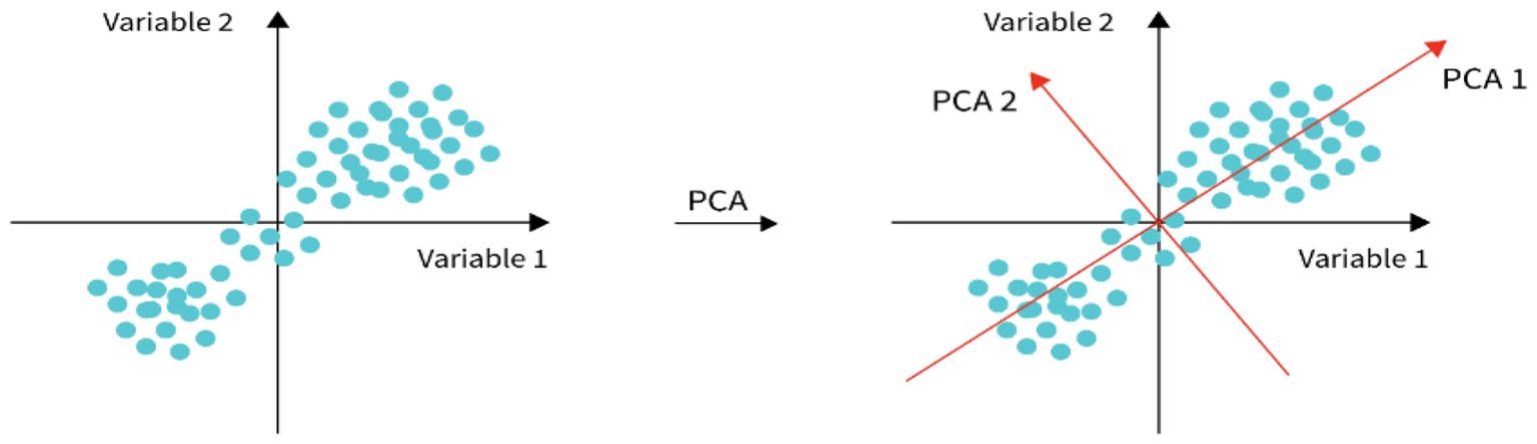
Principal analysis component (PCA) principle.

The ML classification outcomes of 138 oil samples without using the principal component analysis (PCA) are presented in the study. PCA is a well-known feature-harvesting method in mathematical research. This method removes key elements by linear conversion and obtains minimum sizes to demonstrate original data^72^. As a result, PCA is utilized to minimize parameter sizes, remove duplicate data, optimize classifier construction with minimal data loss, as well as enhance classification efficiency^29^. PCA includes the following stages: (i) obtaining the dataset; (ii) normalizing the dataset; (iii) calculating the correlation array; and (iv) interpreting the correlation array^55^. Figure 18 depicts the principle.

The classification learner app utilized for ML classification in MATLAB software provides a diverse set of sub-classifiers. The purpose of integrating these classifiers was to achieve optimal accuracy, and then employ the classifier/s that produce significant results for condition monitoring of the transformer. The results of the classifiers will differ due to some classifiers experiencing overfitting/underfitting as a result of (i) training data size being too small or not containing enough data samples to accurately represent all possible input data values; (ii) when the training data contains large amounts of irrelevant information, known as noisy data; and (iii) when the model trains for too long on a single sample set of data. Moreover, each classifier has different strengths and attributes. The training, validation, and testing process usually takes time, and the amount of time varies depending on the size of the data set and the competence of the classifier. Therefore, the longer it takes to train the classifier, the lower the accuracy, hence the results will be slightly different. The results of the ML classifiers are shown in Table 18. It is worth noting from the results that the highest degree of accuracy is 87.7%, which was produced by Bagged Trees, followed by Fine KNN with 86.2%, and the third in rank is Quadratic SVM with 84.1%.

**Table 18:** ML DGA classification outcomes.

Classifier	Type	Accuracy (%)	Prediction speed (objects/sec.)	Training time (sec.)
DT	Fine tree	82.6	360	79.946
	Medium tree	82.6	360	68.674
	Coarse tree	82.6	390	80.457
SVM	Linear SVM	82.6	53	70.575
	Quadratic SVM	84.1	55	59.946
	Cubic SVM	82.6	71	84.655
	Fine Gaussian SVM	82.6	73	84.433
	Medium Gaussian SVM	82.6	100	103.5
	Course Gaussian SVM	79	100	143.28
KNN	Fine KNN	86.2	230	77.51
	Medium KNN	82.6	240	79.36
	Coarse KNN	77.5	250	159.39
	Cosine KNN	82.6	240	81.82
	Cubic KNN	81.9	350	111.81
	Weighted KNN	81.9	390	111.42
EC	Boosted trees	77.5	540	116.2
	Bagged trees	87.7	34	59.93
	Subspace discriminant	79	38	139.66
	Subspace KNN	81.9	28	64.11
	RUSBoosted trees	82.6	46	67.58

Table 19 illustrates the comparison results of the proposed model and seven DGA approaches, namely: the CIGRE approach, Doernenburg approach, KG approach, Nomograph approach, IEC approach, Duval triangle approach, and Roger's ratio approach. The accuracy of the proposed multi-classification model, consisting of DT, SVM, KNN, and EC is 82.6%, 84.1%, 82.6%, and 87.7% respectively. These were the outcomes of the study. Therefore, when these findings are compared to DGA approaches, they demonstrate a considerable increase in the proportion of accurate fault-type estimation, which is above 20%.

**Table 19:** Comparison between the diagnostic accuracy of several DGA approaches and the proposed multi-classification model for 138 oil samples.

Technique	Correct fault identification (%)
Proposed multi-classification model	82.6; 84.1; 86.2, 87.7
CIGRE approach^123^	80.76
Doernenburg approach^124^	79.03
KG approach^125^	75.8
Nomograph approach^126^	68.69
IEC approach^127,128^	82.25
Duval triangle approach^129,130^	84.11
Roger’s ratio approach^41,131^	71.15

## Conclusions

In this work, a novel multi-classification model that is based on ML algorithms was proposed to have an intelligible, precise, and perfect understanding of DGA. The proposed model was used to analyze 138 TO samples that revealed different stray gassing characteristics in various South African substations. This was achieved by employing four ML classifiers. Experimental evidence using DT classifier viz. Fine Tree, Medium Tree, and Coarse Tree suggested that these classifiers are feasible in classifying stray gas characteristics from normal TO with a degree of accuracy of 82.6%. Additionally, the test results from the SVM classifier comprised Linear SVM, Quadratic SVM, Cubic SVM, Fine Gaussian SVM, Medium Gaussian SVM, and Course Gaussian SVM conclude that these classifiers are viable in classifying stray gassing specificities with the degree of accuracy from 79 to 84.1%. Further, experimental findings from KNN employing Fine KNN, Medium KNN, Coarse KNN, Cosine KNN, Cubic KNN, and Weighted KNN appear to indicate that these classifiers are feasible in classifying stray gassing properties with a degree of accuracy from 77.5 to 86.2%. Lastly, experimental information Boosted Trees, Bagged Trees, Subspace Discriminant, Subspace KNN, and RUS Boosted Trees indicate that these classifiers are feasible in classifying stray gassing peculiarities with a degree of accuracy from 77.5 to 87.7%. The findings can be explained that there are different DG concentrations in stray gassing phenomena from normal TO. In this work, it was demonstrated that the gas concentrations of transformer ${\text{H}}_{2}$, ${\text{C}}{\text{H}}_{4}$,${\text{CO}}$, ${\text{C}}_{2}{{\text{H}}}_{4}$, and ${\text{C}}_{2}{{\text{H}}}_{6}$ can be used to discriminate stray gassing phenomena from normal TO and their differences can be classified with the highest degree of accuracy of 87.7% by the Bagged Trees classifier over other ML classifiers. It is advisable that the training, testing, and validation of DGA oil samples be extended and tested several times to validate the findings in this work.

For forthcoming studies, the findings amassed in this work can be employed as a benchmark in developing a portable device that utilizes ML algorithms herein for the validation of DGA results. Another proposal is that additional research is undertaken in applying DTs to formulate new stray gassing limits for the various gas concentrations. In this fashion, the production of DGs will be easily interpreted with proper permissible levels thereby utility owners and consequently, consumers do not have to suffer from prolonged power outages. Furthermore, the authors will test the proposed model based on ANN algorithms and compare it to the results presented in this work to determine which approach yields more accurate statistics.

## Data Availability

The data that support the findings of this study are available from the corresponding author upon reasonable request.
